# Supplementary material for: Schistosomiasis screening in non-endemic countries from a cost perspective: Knowledge gaps and research priorities. The case of African long-term residents in a Metropolitan Area, Spain
Source: PLoS Negl Trop Dis. 2023 Apr 4;17(4):e0011221. doi: 10.1371/journal.pntd.0011221 (PMC10104311; doi:10.1371/journal.pntd.0011221)
Supplement: S1 Text — Table A in S1 Text: Mean expected estimated cost for each schistosoma-associated pathology and occurrence proportion. Tables B in S1 Text: Estimated averted costs with TT strategy with 2 PZQ dosage. In the y axis the estimated rate of cure/estimated prevalence of the infection in the targeted population. Shadowed in grey we emphasize the scenarios with a negative results. Averted costs are expressed in Euros. Table C in S1 Text: Estimated averted costs with TT strategy with 2 PZQ dosage. In the y axis the estimated rate of cure/estimated prevalence of the infection in the targeted population. Shadowed in grey we emphasize the scenarios with a negative results. Averted costs are expressed in Euros. Table D in S1 Text: Estimated averted costs with PT strategy with 1 PZQ dosage. In the y axis the estimated rate of cure/estimated prevalence of the infection in the targeted population. Shadowed in grey we emphasize the scenarios with a negative results. Averted costs are expressed in Euros. Fig A in S1 Text: Theoretical averted costs under different scenarios of Schistosoma prevalence, long-term morbidities-associated costs and treatment efficacy for PT and TT strategies and WW strategy*. *Dashed horizontal line indicates the zero balance. (DOCX) [file pntd.0011221.s001.docx]

**Table A**: Mean expected estimated cost for each schistosoma-associated pathology and occurrence proportion

|  | **Pathology** | **Mean expected cost (€) *** | **Estimated Proportion of occurrence (%)** | **Weighted cost (€)** | **% w.r.t total** |
| --- | --- | --- | --- | --- | --- |
|  | Hepatomegaly | 542 | 15.7 ^1^ | 85 | 2.85% |
|  | Blood in the stool | 581 | 8.1 ^1^ | 47 | 1.57% |
|  | Splenomegaly | 455 | 11.7 ^1^ | 53 | 1.78% |
|  | Ascites | 3,016 | 0.5 ^1^ | 16 | 0.53% |
|  | Hematemesis | 4,89 | 1.7 ^1^ | 84 | 2.81% |
|  | Hematuria | 485 | 63.4 ^1^ | 307 | 10.25% |
|  | Bladder cancer | 20,371 | 2.72 | 550 | 18.36% |
|  | Dysuria/minor bladder morbidity | 538 | 67.9 ^1^ | 365 | 12.18% |
|  | Major bladder morbidity | 900 | 21.4 ^1^ | 193 | 6.43% |
|  | Hydronephrosis/urethral stenosis | 463 | 8.6 ^1^ | 40 | 1.33% |
|  | Severe kidney failure | 4,872 | 1.5 ^1^ | 74 | 2.47% |
|  | Female infertility | 6,012 | 15.0^1^ | 902 | 30.10% |
|  | Stroke | 6,425 | 4.004^2^ | 161 | 5.36% |
|  | Myelitis | 2,774 | 0.1^2^ | 3 | 0.09% |
|  | Female genital schistosomiasis | 307 | 33.3^1^ | 102 | 3.41% |
|  | Glomerulonephritis | 701 | 0.9^2^ | 7 | 0.22% |
|  | Pulmonary hypertension | 2,361 | 2.6^2^ | 61 | 2.04% |

*Cost source: Hospital Universitari Germans Trias i Pujol (see data availability statement).* 1*.*Based on [1,2]. 2 Based on [3]

**Tables B** : Estimated averted costs with TT strategy with 2 PZQ dosage. In the ***y*** axis the estimated rate of cure/estimated prevalence of the infection in the targeted population. Shadowed in grey we emphasize the scenarios with a negative results. Averted costs are expressed in Euros.

|  | | |  | |  | |  | |  | |  | |  | |  | |  |
| --- | --- | --- | --- | --- | --- | --- | --- | --- | --- | --- | --- | --- | --- | --- | --- | --- | --- |
| **TT-2** | ***30%*** | | ***40%*** | | ***50%*** | | ***60%*** | | ***70%*** | | ***80%*** | | ***90%*** | | ***100%*** | |  |
| ***100/30%*** | | 6008644 | | 11390859 | | 16773074 | | 22155289 | | 27537503 | | 32919718 | | 38301933 | | 43684148 | |
| ***100/24%*** | | 3711715 | | 8017487 | | 12323259 | | 16629031 | | 20934803 | | 25240574 | | 29546346 | | 33852118 | |
| ***100/15%*** | | 266322,1 | | 2957430 | | 5648537 | | 8339644 | | 11030752 | | 13721859 | | 16412966 | | 19104074 | |
| ***75/30%*** | | 1971983 | | 6008644 | | 10045305 | | 14081966 | | 18118627 | | 22155289 | | 26191950 | | 30228611 | |
| ***75/24%*** | | 482386,6 | | 3711715 | | 6941044 | | 10170373 | | 13399702 | | 16629031 | | 19858360 | | 23087689 | |
| ***75/15%*** | | -1752008 | | 266322,1 | | 2284653 | | 4302983 | | 6321314 | | 8339644 | | 10357975 | | 12376305 | |
| ***50/30%*** | | -2064678 | | 626429,5 | | 3317537 | | 6008644 | | 8699752 | | 11390859 | | 14081966 | | 16773074 | |
| ***50/24%*** | | -2746942 | | -594056 | | 1558830 | | 3711715 | | 5864601 | | 8017487 | | 10170373 | | 12323259 | |
| ***50/15%*** | | -3770339 | | -2424785 | | -1079232 | | 266322,1 | | 1611876 | | 2957430 | | 4302983 | | 5648537 | |

**Table C:** Estimated averted costs with TT strategy with 2 PZQ dosage. In the y axis the estimated rate of cure/estimated prevalence of the infection in the targeted population. Shadowed in grey we emphasize the scenarios with a negative results. Averted costs are expressed in Euros.

| **TT-1** | ***30%*** | ***40%*** | ***50%*** | ***60%*** | ***70%*** | ***80%*** | ***90%*** | ***100%*** |
| --- | --- | --- | --- | --- | --- | --- | --- | --- |
| ***100/30%*** | 7784644 | 13166859 | 18549074 | 23931289 | 29313503 | 34695718 | 40077933 | 45460148 |
| ***100/24%*** | 5132515 | 9438287 | 13744059 | 18049831 | 22355603 | 26661374 | 30967146 | 35272918 |
| ***100/15%*** | 1154322 | 3845430 | 6536537 | 9227644 | 11918752 | 14609859 | 17300966 | 19992074 |
| ***75/30%*** | 3747983 | 7784644 | 11821305 | 15857966 | 19894627 | 23931289 | 27967950 | 32004611 |
| ***75/24%*** | 1903187 | 5132515 | 8361844 | 11591173 | 14820502 | 18049831 | 21279160 | 24508489 |
| ***75/15%*** | -864008 | 1154322 | 3172653 | 5190983 | 7209314 | 9227644 | 11245975 | 13264305 |
| ***50/30%*** | -288678 | 2402430 | 5093537 | 7784644 | 10475752 | 13166859 | 15857966 | 18549074 |
| ***50/24%*** | -1326142 | 826743,6 | 2979630 | 5132515 | 7285401 | 9438287 | 11591173 | 13744059 |
| ***50/15%*** | -2882339 | -1536785 | -191232 | 1154322 | 2499876 | 3845430 | 5190983 | 6536537 |

**Table D:** Estimated averted costs with PT strategy with 1 PZQ dosage. In the ***y*** axis the estimated rate of cure/estimated prevalence of the infection in the targeted population. Shadowed in grey we emphasize the scenarios with a negative results. Averted costs are expressed in Euros.

| **PT** | ***30%*** | ***40%*** | ***50%*** | ***60%*** | ***70%*** | ***80%*** | ***90%*** | ***100%*** |
| --- | --- | --- | --- | --- | --- | --- | --- | --- |
| ***100/30%*** | 6526644 | 11908859 | 17291074 | 22673289 | 28055503 | 33437718 | 38819933 | 44202148 |
| ***100/24%*** | 3297315 | 7603087 | 11908859 | 16214631 | 20520403 | 24826174 | 29131946 | 33437718 |
| ***100/15%*** | -1546678 | 1144430 | 3835537 | 6526644 | 9217752 | 11908859 | 14599966 | 17291074 |
| ***75/30%*** | 2489983 | 6526644 | 10563305 | 14599966 | 18636627 | 22673289 | 26709950 | 30746611 |
| ***75/24%*** | 67987 | 3297315 | 6526644 | 9755973 | 12985302 | 16214631 | 19443960 | 22673289 |
| ***75/15%*** | -3565008 | -1546678 | 471653 | 2489983 | 4508314 | 6526644 | 8544975 | 10563305 |
| ***50/30%*** | -1546678 | 1144430 | 3835537 | 6008644 | 9217752 | 11908859 | 14599966 | 17291074 |
| ***50/24%*** | -3161342 | -1008456 | 1144430 | 3297315 | 5450201 | 7603087 | 9755973 | 11908859 |
| ***50/15%*** | -5583339 | -4237785 | -2892232 | -1546678 | -201124 | 1144430 | 2489983 | 3835537 |

**Figure A**: Theoretical averted costs under different scenarios of *Schistosoma* prevalence, long-term morbidities-associated costs and treatment efficacy for PT and TT strategies and WW strategy*


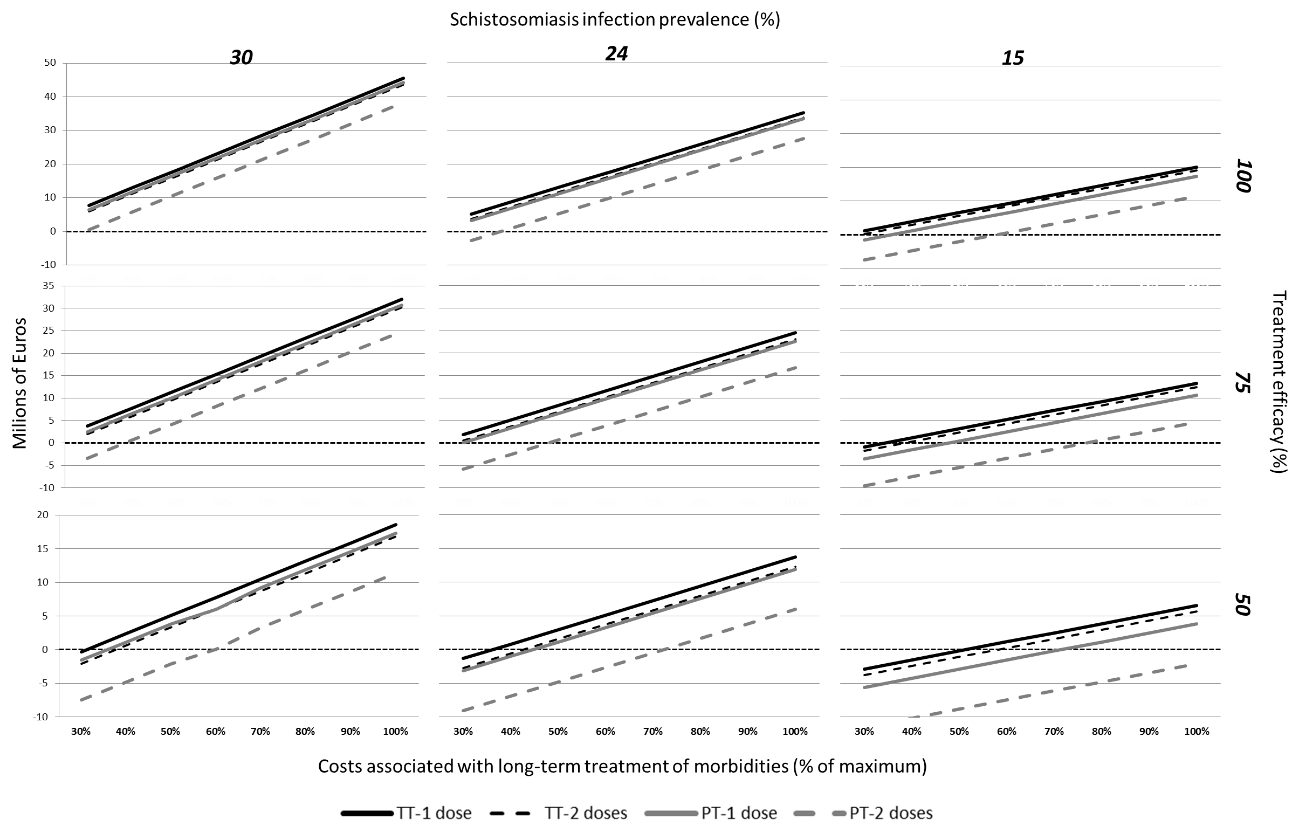


*Dashed horizontal line indicates the zero balance.

**References**

1. *World Health Organization Expert Committee on the Control of Schistosomiasis. Public health impact of schistosomiasis: disease and mortality. Bull World Health Organ. 1993;71:657-62.*
2. *World Health Organisation. Current estimated total number of individuals with morbidity and mortality due to Schistosomiasis Haematobium and S. Mansoni infection in Sub-Saharan Africa. Available at* [*https://www.who.int/images/default-source/departments/ntd-library/schistosomiasis/estimated-morbidity-and-mortality-due-to-schistosomiasis.png?sfvrsn=c04e610f_0*](https://www.who.int/images/default-source/departments/ntd-library/schistosomiasis/estimated-morbidity-and-mortality-due-to-schistosomiasis.png?sfvrsn=c04e610f_0)*. Last access May 2022*
3. *Webb JA, Fabreau G, Spackman E, Vaughan S, McBrien K. The cost-effectiveness of schistosomiasis screening and treatment among recently resettled refugees to Canada: an economic evaluation. C. open, 2021; 9 : E125–E133. doi: 10.9778/cmajo.20190057.*
