# Supplementary material for: Schistosomiasis screening in non-endemic countries from a cost perspective: Knowledge gaps and research priorities. The case of African long-term residents in a Metropolitan Area, Spain
Source: PLoS Negl Trop Dis. 2023 Apr 4;17(4):e0011221. doi: 10.1371/journal.pntd.0011221 (PMC10104311; doi:10.1371/journal.pntd.0011221)
Supplement: Attachment — Submitted filename: Answer_reviewers_1210.docx [file pntd.0011221.s003.docx]

We would like to thank sincerely the suggestions and ammendments proposed by the reviewers, which have help to improve the article. First , we introduced few general ammendments in the article that may address some of the questions raised by them.

1. At the end of the introduction section, page 7, lines 145-152, we introduced the following paragraph:

“Our study aims to raise the gaps in current knowledge regarding a variety of issues impacting the cost of schistosomiasis screening program in non-endemic countries. Based on these observations, we build a simple static cost analysis comparing a watchful waiting approach, currently the status quo in most non-endemic countries, with either presumptive treatment or test-and-treat strategies, considering different reasonable scenarios and necessary assumptions. Finally, the analysis allows us to points out the areas where further research is necessary.”

2. We included an introductory paragraph at the beginning of the Material and Methods section which explanis step by step de analysis process (page 8, lines 155-172):

“This is a four steps analysis. At first instance, in order to identify factors that might influence the costs and costs/benefit ratio of a schistosomiasis screening program, we hosted a round table discussion that included health professionals working in the area of imported diseases and/or migrant populations and health economists in our study area. For each such factor identified, we then carried out a narrative review of the current literature. We determined the realistic bounds of factors which could reasonably be estimated among our study population (i.e. prevalence of infection) to be applied to our model. At second instance, we develop a modelling economic study with a static approach based on our study population, considering different scenarios and assumptions according to the previous assessment, comparing three strategies (wait and watchful, presumptive treatment and test and treat). Thirdly, we confronted the model with additional identified co-factors from which we could not yet ascertain reasonable bounds estimations and should be subject to further research or evaluation (see results section). Finally, we develop a list of research priorities raised by our analysis, in order to determine or develop the most suitable schistosomiasis-screening strategy of imported schistosomiasis in non-endemic countries (see discussion section).“

3. We moved the section “Main factors that might influence to the costs associated with Screening strategies” after the introductory pargraph in the Materials and Methods section. This includes the factors from which reasonable bounds could be estimated and are considered in the model.

4. We added a number of new references, including some suggested by the reviewers, that we mention below.

5. We removed from the analysis and the text the Presumptive treatment with 2 dosages (PT-2), which has been considered unrealistic for a presumptive treatment strategy.

6. We included some additional sentences/paragraphs:

Discussion, page 28, lines 499-503: “These observations point out first, that any screening program should include the active referral and linkage to care of patients with particular clinical manifestations for adequate follow-up, and second, that the program should aim to reach recently arrived migrants where higher cost/benefit and treatment efficacy would be expected.”

Discussion, page 29, lines 570-574: “Finally, the high rate of lost-of follow-up of patients under schistosomiasis-infection evaluation observed in our study region [13] and other sites [12], supports a community-based catch and treat option (PT or TT) even with a single dosage to ensure the effective PZQ intake. With this regard, a POC would be most appropriated for a TT strategy.”

7. Former table 3 have been moved to supplementary material (Table S1). We added to the supplementary material tables S2-S4, which show the detailed averted costs under different explored scenarios. Figure 3 have been included as supplementary material (now Figure S1). A dynamic table with the baseline data used for calculations have been included, as well.

Below you will find the specific answers to the questions and concerns raised by the reviewers:


Reviewer #1: -The objectives are to compare theoretically 3 strategies and their cost against imported schistosomiasis (spontaneous evolution, presumptive teatment, test and treat if +); to look at factors influencing the benefits of each strategy. Assumptions set-up for the decision analysis model are presented.
It is an interdisciplinary study with a valuable insight in the approaches of schistosomiasis management in a South european country, one of the main "entry gate" of Sub-Saharan immigrants in Europe.
-The question of serious morbidity detection in the management of Schistosomiasis screening appears understated.

**Answer:** Certainly, one of the key message we want to underscore from our analysis is the lack of systematic screening programs in Europe and the uwareness of the potential high burden of schistosomiasis-related morbidity in sites with a substantial pockets of migrant populations. This has been emphasized in the following sentences:

Introduction, page 6, lines 120-122: “The impact of schistosomiasis on public health systems in non-endemic regions like Europe and North America has risen lately due to increasing global migration from endemic regions, mostly sub-Saharan Africa [7,8].”

Introduction, page 6-7, lines 125-137: “However, Schistosoma screening programs are scarce in Europe and clinical suspicion of the infection very low, in spite of the recommendation by the ECDC to do so in all arriving migrants [10] and the evidences collected so far[11] . Late diagnostic of schistosomiasis infection and associated morbidities seems to be the rule [11-13]. This means that many cases may pass undetected, leading to the development of long-term chronic complications with concomitant high care-associated costs (e.g. dialysis for patients with terminal renal insufficiency) [11, 14,]. The main issue about laboratory methods is the lack of specificity towards active infection (serology-based kits) and lack of sensitivity of urine/stool examination in non-endemic countries [15, 16]. Overall, this may lead to an unknown prevalence of Schistosomiasis infection and associated morbidity especially among long-term resident migrants. “

Footnote table 1: “The real prevalence of the active infection in long-term migrants in Europe remains unknown.”

-The population described live in a sizable single area which welcomes a high number of immigrants and where concentrate primary care facilities: 74 000 people estimated to be exposed to chronic infection. However, socio-demographic characteristics are not detailed.

**Answer:** We included the following additional information:

Materials and Methods, Page 10, lines 213-217: “The mean age of migrant population in our study area is 34,3 yrs. substantially younger than locals (44,4 yrs.) [18] and 32.7% come from Senegal (see the list of main countries of origin in Table 1). . According to our own records 63% have been living in our country form more than 10 years [13] “

-Authors present with honesty the numerous knowledge gaps that limit significantly the results, especially the factors influencing each strategy. 
-No ethical concern.

Reviewer #2: (No Response)

--------------------

<b>Results</b></br></br>
-Does the analysis presented match the analysis plan?</br>
-Are the results clearly and completely presented?</br>
-Are the figures (Tables, Images) of sufficient quality for clarity?</br></br>

Reviewer #1: The analysis plan lack of solid assumptions on certain points: prevalence of infection, prevalence of serious morbidity, screening method.

Authors could have presented a Table listing the assumptions in each strategy with their respective evidences FOR and AGAINST them.

**Answer:** Our analysis doesn’t attempt to provide a comprehensive cost-economic analysis but to underscore the factors that hamper such attempt. With this regard, we applied a simplistic model using diferent assumptions/scenarios according to the available evidence, and discuss the results in light of the knowledge gaps. However, we included a short table with assumptions FOR and AGAINST as suggested by the reviewer as follows (Now Table 4) at the discussion section (page 30):

Table 4: For and against each strategy according to different considered assumptions.

| Strategy | For | Against |
| --- | --- | --- |
| Wait and watchful strategy | Lack of efficacy of Schistosomiasis treatment over long-term conditions | PZQ Treatment at early stages of a given chronic complication may result in a better prognostic |
|  |  |  |
|  |  |  |
|  | Low prevalence of the infection |  |
| Presumptive treatment | Easier access to hard-to-reach populations at risk | No specific diagnostic would be made |
|  | Lack of sensitive and specific diagnostic tools | Low coverage |
|  | Access to hard-to-reach populations | Lack of follow-up of patients |
|  | High effective in recently arrived migrants from endemic countries | Risk of a high number of unnecessary treatments with secondary effects |
|  |  |  |
|  | High prevalence of infection |  |
| Test and treat | More specific and targeted treatment | Lack of specific and sensitive diagnostic tests and screening procedures |
|  |  |  |
|  | Availability of sensitive and specific diagnostic tools |  |
|  |  |  |
|  | Allows to follow-up patients and evaluate treatment efficacy |  |
|  |  |  |
|  | High-middle prevalence of the infection | Low coverage |
|  |  |  |

Although it is specified that results apply to the local situation of Barcelona, some of the assumptions are drawn not from local or national studies, even though such data exist. Concerning the prevalence of infection, it is estimated by a pooled seroprevalence from international surveys, some conducted quite a long time ago (Asundi et al.). Authors could have extracted from this paper Spanish or South-European study data reviewed, in order to get a sharper figure.

**Answer:** We would like to point out that the published studies in Europe are carried out among populations with similar background than our study population (i.e. similar countries of origin). With this regard, our study population are mostly long-term residents from which old estimations may still be valid. Otherwise, community based data about the sero-prevalence of schistosomiasis infection in our region is scarce. The most comprehensive data published to date comes from our own records which has been referenced (refs. 13 and 38), which are similar than the observed from other Spanish teams (refs. 12, 44 and 45). The lack of certitude about the truly prevalence of Schistosomiasis in our population has been overcome considering diferent scenarios of prevalence (between 5 to 50%), considering as a baseline reference 24% . Prevalence assumption has been considered as the existence of schistosomiasis infection susceptible to respond to the PZQ treatment. To our knowledge, these are the best estimates we may have. Otherwise, we clarify as sero-prevalence, not prevalence whenever adequate throughout the text and we make it clearer that sero-prevalence may not correlate exactly with prevalence of the infection as follows:

Introduction, page 7, lines 133-137: “The main issue about laboratory methods is the lack of specificity towards active infection (serology-based kits) and lack of sensitivity of urine/stool examination in non-endemic countries [15, 16]. Overall, this may lead to an unknown prevalence of Schistosomiasis infection and associated morbidity especially among long-term resident migrants. “

Material and methods section, page 9, lines 182-185: “However, the correlation between serology (sero-prevalence) and active infection susceptible to respond to specific treatment (real prevalence) is far from clear, specifically among long-term migrants.”

Footnote Table 1: “These estimations correspond to the observation of eggs in urine or stool samples and mostly coming from children or young adults, whereas the estimations in Europe are from sero-prevalence studies . The real prevalence of the active infection in long-term migrants in Europe remains unknown”

Indeed, infection prevalence (not seroprevalence) depends closely on the sociodemographic profile of the immigrants in the place they settled: age, sex, geographic origin... at the village level, because of the overdispersed distribution of Schistosomes among populations and age groups.

**Answer:** We fully agree with your consideration. In the discussion section we mention that any screening program would likely be more or less suitable depending on targeted sub-populations among exposed migrants. We mention this consideration in the discussion section:

Page 32, lines 615-623: “Consequently, a more comprehensive cost analysis should consider different subsets of exposed populations, each of which may require a tailored intervention and associated use of resources. Such population subset studies might look at long-term resident migrants vs. recent migrants, infants, females, specific countries of origin, geographic regions or even villages or second generation migrants born in Europe that travel regularly to their family’s country of origin. The PT strategy might be the most optimal option in a specific subset of these populations (e.g. those with more recent infections and highest prevalence).”

Unfortunately, we could only determine the risk at country-level origin, but we aim in an ongoing study to include the village of origin of participants to ascertain the risk by more specific geographic origin and village. This complementary information would certainly guide the screening program applied in the near future.

The other inconvenient is that it is a seroprevalence which overestimates the real situation. The fact that it corresponds to Schisto prevalences (mostly based on microscopic examination) in the six African countries most represented in the immigrant community of Barcelona seemed to be a coincidence.

**Answer**: We fully agree with your consideration that seroprevalence may overestimate the real situation. However, our current data suggest that a substantial number of exposed individuals with a serology-negative results present signs and symptoms compatible with long-term schistosomiasis-associated morbidity (see reference 13). In other words, serology results could not roll out the presence or absence of schistosomiasis infection or associated-morbidities. This is the main limitation of the current diagnostic tools in non-endemic countries since urine/stool examination have a very poor sensitivity. Consequently, one of the main priority research areas suggested in our conclusions is the development of new diagnostic tools with higher sensitivity and specificity. We underpine this in the following sentences,

Results section, page 23-24, lines 440-443: “Otherwise, a serology negative results could not roll out the existence of infection and consequently, the clinical evaluation of those patients with signs and symptoms compatible with Schistosoma infection [13].”

Discussion section, page 27-28, lines 523-527: “The lack of sensitivity/specificity of serology test for the TT strategy could be overcome by the inclusion of a check list of signs and symptoms as we suggested previously [13] or three steps combining serology, microscopy examination and ultrasound or clinical assessment [17]”

Discussion section, page 31, lines 588-594: “Future research should also focus on the development of point-of-care (POC) tests ( e.g. a finger-prick blood test) [50] that would help to effectively implement a rapid test-and-treat strategy, and the development of more specific diagnostic methods in order to avoid unnecessary treatment (e.g. a PCR-based diagnostic that could differentiate among Schistosoma species, for clinical and/or laboratory use) to overcome the limitations of the serology test (including false-negative results).”

The coincidence between the estimated prevalences and the prevalence in our study population could not be regarded as this. As the reviewer rightly observe the prevalence in countries of origin are based on urine/stool examination and most probably among children and young people whereas our prevalence is an indirect estimation through sero-prevalence among long-term migrants. We included this observation as a foot-note in Table 1 as follows:

“These estimations correspond to the observation of eggs in urine or stool samples and mostly coming from children or young adults, whereas the estimations in Europe are from sero-prevalence studies . The real prevalence of the active infection in long-term migrants in Europe remains unknown”

In the first scenario (Watchfull waiting), assumptions concerning the organ involvements in Schistosomiasis (serious morbidity) are rare, although the primary issue of the  study is to prevent or reduce this occurence after it has been detected. In this light, a body of a dozen publications on the subject in non endemic European countries are lacking as references: about 4 are Spanish, 4 or 6 are from Italy, 2 or 3 come from France. About the rate of hospitalization mentioned in Table 2, the EurotropNet surveillance datareported a 11 % rate among migrants diagnosed with positive urine microscopy (Lingscheid et al.).

**Answer:** Thank you for your suggestion. We agree that serious morbidity occurrence are estimated as rare. However, recent studies in Spain points out that as high as 4% of newly diagnostics presented a serious disease (reference 47) . Furthermore, the ageing of the exposed population means that probably we are not yet observing the full array of such complications. Recent estimates indicate a high prevalence of Female Genital Schistosomiasis (reference 41). In our understanding we don’t know exactly the burden or potential burden of schistosomas-associated disease in non-endemic countries. This is one of the areas which deserve urgent attention. After revision of the suggested references, we included the following statements in the discussion section:

Page 28, lines 526-530: “Otherwise Marchese et al. found a 92% sero-prevalence and 38% of microscopy diagnostic among 272 diagnosed patients [46] and Salas-Coronas found a prevalence of 10.5% [47], but the time of exposure was lower than one year and around 3 years, respectively. “

page 29, lines 559-563.: “An Spanish series found a 4% prevalence of severe morbidity among patients with confirmed infection [47], which may increase over time given the chronic nature of the infection. Hospitalization and chronic symptoms have been found highly prevalent among imported schistosomiasis infections in Europe [48]. “

...alongside the inclusion in the reference list of these new references:

46.	Marchese V, Beltrame A, Angheben A, Monteiro GB, Giorli G, Perandin F et al. Schistosomiasis in immigrants, refugees and travellers in an Italian referral centre for tropical diseases. Infect Dis Poverty. 2018;7:55. doi: 10.1186/s40249-018-0440-5.

47.	Salas-Coronas J, Vázquez-Villegas J, Lozano-Serrano AB, Soriano-Pérez MJ, Cabeza-Barrera I, Cabezas-Fernández MT et al. Severe complications of imported schistosomiasis, Spain: A retrospective observational study. Travel Med Infect Dis. 2020;35:101508. doi: 10.1016/j.tmaid.2019.101508.

48.	Lingscheid T, Kurth F, Clerinx J, Marocco S, Trevino B, Schunk M et al; TropNet Schistosomiasis Investigator Group. Schistosomiasis in European Travelers and Migrants: Analysis of 14 Years TropNet Surveillance Data. Am J Trop Med Hyg. 2017;97:567-574. doi: 10.4269/ajtmh.17-0034.

As for assumptions, estimated frequency of symptoms of infection, s. of complication, relative part of Schisto species among the popultion studied (about 80 S. haem./20 S. mansoni ?), estimated effect of praziquantel on regression of organ involvement, accessibility of migrants to primary care facilities, estimated under-diagnosis in these settings, etc.
In fact, authors chose to present some of these latters among "Co-factors that may influence the cost-benefit ratio" (p. 20). The choice is respectful but some should appear before, among the assumptions presentation, the 2 most important in the Watchfull Waiting scenario being that:
-ultrasonography is the gold standard to detect organ involvements
 -ultrasonography abnormalities considered to be related to such involvements are detected significantly more often in case  of positive microscopic examination of urine than in case of positive serology. It seems it has not been assessed for intestnal schistosomiasis.
An recent Italian paper presenting evidences of regression of bladder involvements concludes "Mucosal masses in young patientsregressed after treatment without recurrence, supporting the recommendation that invasive procedures should be avoided unless lesions or other signs of concern persist for > 6 months."

**Answer:** We agree with the observations raised by the reviewer. Accordingly, we reshaped the methods section (see at the beginning of this response letter), to make it clear that the assumptions are those from which a reliable scenarios of proportion/prevalence could be estimated. In a second step, we discuss other co-factors from which we are unable to estimate the true effect towards cost-effectiveness.

More precisely, in page 21, line 376 of the results section we specified as a factor that may impact on the cost and cost benefit ratio “the regimen and dosage of PZQ”, which is further developed.

We would like to point out that the mentioned italian article includes young (mean age of 26 yrs.) and newly arrived exposed patients (less than 5 years), which do not correspond to our long-term exposed-migrant profile which are older and use to have spent moret han 10 yrs in our country. However, we included the following statement alongside this new reference (now reference 31: Tamarozzi F, Ursini T, Ronzoni N, Badona Monteiro G, Gobbi FG, Angheben A et al. Prospective cohort study using ultrasonography of Schistosoma haematobium-infected migrants. J Travel Med. 2021;28:taab122. doi: 10.1093/jtm/taab122.): Results section, page 22, lines 396-399: “It is also not yet clear how PZQ administration impacts chronic lesions (i.e. eggs-diposit induced fibrosis); according to some reports bladder lesions and fibrosis reverses after therapy, but advanced pathology may persist for a long time in spite of treatment or non-reexposure [31, 32].”

Otherwise The ultrasonography procedures for the WW strategy and associated costs have been taken into account in the costs-calculations (see Table S1, supplementary material).

The second scenario (Presumptive treatment) could be assumpted not only by studies on praziquantel efficacy, but acceptability studies of such administration. People usually prefer to take a medicine for an identified cause rather than "in case of ...".

**Answer:** We agree with your suggestion. We mention the acceptability issue in the Results section, page 23, lines 420-427:

“Consequently, the implementation of a PT or TT strategy would ideally require the allocation of resources for active-case detection and outreach activities, with the provision of the necessary procedures recommended for health promotion programs in Europe, such as voluntary, confidential and non-stigmatizing screening and efficient linkage to appropriate care and treatment [39] as well as awareness-raising programs to increase the acceptability of screening and treatment among targeted communities.”

And we included the following statement in the Discussion section, page 32, lines 622-627:“With this regard, acceptability studies seems to be most appropriated prior the implementation of an screening program”

One important assumption of this scenario is the number of doses of praziqantel. If we empirically think that 2 would be more efficient than one, at which time interval should they be delivered ? How many persons with organ involvement would not be cured enough with 2 doses ?.. an ethical question raised by this scenario.

**Answer:** This issue is clearly raised and discussed in point 2) of co-factors that may influence the costs of an screening program: “2) Treatment regimen and dosage”. We included at the end of this paragraph the following additional reflection:

Results section, page 22, lines 407-411:“ These observations point out first, that any screening program should include the active referral and linkage to care of patients with particular clinical manifestations for adequate follow-up, and second, that the program should aim to reach recently arrived migrants where higher cost/benefit and treatment efficacy would be expected.”

We included a couple of new references to expand the issue about the dosage of PZQ in schistosomiasis chronic infections:

28.	Zwang J, Olliaro PL. Clinical efficacy and tolerability of praziquantel for intestinal and urinary schistosomiasis-a meta-analysis of comparative and non-comparative clinical trials. PLoS Negl Trop Dis. 2014;8:e3286. doi: 10.1371/journal.pntd.0003286.

30.	Cucchetto G, Buonfrate D, Marchese V et al. High-dose or multi-day praziquantel for imported schistosomiasis? A systematic review. J Travel Med. 2019;26:taz050. doi: 10.1093/jtm/taz050.

...alongside the following statements in the Results section:

Page 21-22, lines 386-389: “…As a result, though the current guidelines in our study area advise the administration of PZQ to any suspected infection based on serology test [10], the baseline evidence of efficacy is still lacking specially among long-term infections [28].”

Page 22, lines 392-396: “The optimal treatment efficacy cut-offs are still a matter of debate, including dosage regimen, and multicenter clinical trials in non-endemic areas are still needed [30], but it seems clear that a single dose does not reach 100% efficacy in the chronic phase [28]”

The third scenario (Test and treat) is based on serology testing. As previously mentioned, positive serology is not significantly associated with organ involvements.

**Answer:** The screening program aims primarily to prevent the development of an organ involvement and chronic conditions through early treatment. The question is whether an stand-alone serology-test would not detect a substantial number of individuals that may benefit of PZQ treatment or with long-term conditions susceptible to improve with PZQ administration. This is an important gap of knowledge that we underline. With this regard, we mention that the test and treat scenario would need a clinical check-list as suggested by our previous publication and the Australian guidelines (now reference 17) , alongside a referral for patients with concerning clinical simptoms. We mention theses issues as follows:

Discussion section, page 27-28, lines 520-524: “The lack of sensitivity/specificity of serology test for the TT strategy could be overcome by the inclusion of a check list of signs and symptoms as we suggested previously [13] or three steps combining serology, microscopy examination and ultrasound or clinical assessment [17]”

Discussion section, page 29, lines 557-559: “With this perspective, the TT strategy including a check list of signs and symptoms, would allow the inclusion of a referral and linkage to care system for patients suspected of serious morbidities which need a closer follow-up.”

Moreover, it can give false negative results, an outcome representing a loss of chance in matter of health to people in this case with organ involvement.

**Answer:** See our previous answer regarding the necessity of referral for patients with suspected symptoms and the issue about false negative results.

In addition it is not as relevant in detecting Schisto infection in a population group where prevalence of infection is high. As we don't know precisely the prevalence in Barcelona, the choice may be risky.

**Answer:** We fully agree with your appreciation. The lack of highly specific and sensitive diagnostic tools and means that we don’t know truly prevalence of schistosomiasis infection/morbidities among long-terme migrants. Our modelling results clearly indicates that the best choice screening method depend on infection prevalence. However, all data available strongly suggest that the prevalence is high enough to foresee either a TT or PT strategy in our region. We think these statements have been well developed in the article, as you can see in our previous answers.

Unfortunately authors don't suggest alternatives to serology that could be assessed.

**Answer:** We suggest the inclusion of clinical check list as we mention above and the development of rapid serology or antigen-detection kits, that will allow to stablish an POC strategy, as follows:

Discussion section, pages 30-31, lines 585-591: “Future research should also focus on the development of point-of-care (POC) tests ( e.g. a finger-prick blood test) [51] that would help to effectively implement a rapid test-and-treat strategy, and the development of more specific diagnostic methods in order to avoid unnecessary treatment (e.g. a PCR-based diagnostic that could differentiate among Schistosoma species, for clinical and/or laboratory use) to overcome the limitations of the serology test (including false-negative results)”

It is a pity that microcopic examination is not mentioned in the paper, even if it is not a good screening method, theoretically. Some papers (not mentioned in the references) explore the feasibility of screening procedures using clinical checklist and serology, or a step by step clinical assessment with urine dipstick in the detection of S. haematobium. The Australian health Authorities foresee a parasitic examination of stools and urine among migrans from endemic regions, after a positive serology, alongside the cure.

**Answer:** Certainly, the use of microscopic examination should not be excluded but it is not feasible during an community-based screening with a test-and-treat approach, besides the very low sensitivity in non-endemic countries. We state this last appreciation in the results section, page 21, lines 383-388: “However, the excretion of eggs in long-term exposed adult patients (like most infected migrants) is rarely observed in non-endemic countries and thus PZQ efficacy cannot be evaluated in these terms. As a result, though the current guidelines in our study area advise the administration of PZQ to any suspected infection based on serology test [10],.”

We mention as well the Australian guideline which propose the use of urine examination, alongisde a serology and clinical screening. However, we would like to point out that our study population are mostly long-term residents from which the urine/stool examination have a very poor yield, whereas the study from Australia considers new arrivals which have a much higher yield of direct examination. In this last case the use of urine/stool examination makes lot of sense. We include the following sentence in the discussion section:

Pages 27-28, lines 520-526: “The lack of sensitivity/specificity of serology test for the TT strategy could be overcome by the inclusion of a check list of signs and symptoms as we suggested previously [13] or three steps combining serology, microscopy examination and ultrasound or clinical assessment [17]. However, the inclusion of urine/stool examination proposed by the Australian health authorities have been designed for recent arrived migrants, but not for long-term migrants.”

These "two step" procedures may be worth to be assessed, even as screening procedures.

**Answer:** Sorry for not being clearer. We introduced the following sentences to suggest that an stand-alone serology screening might be insuficient and needs a two-step procedure using clinical+serology:

Discussion section, pages 27-28, lines 520-526: “The lack of sensitivity/specificity of serology test for the TT strategy could be overcome by the inclusion of a check list of signs and symptoms as we suggested previously [13] or three steps combining serology, microscopy examination and ultrasound or clinical assessment [17]”

Discussion section, page 29, lines 557-559: “With this perspective, the TT strategy including a check list of signs and symptoms, would allow the inclusion of a referral and linkage to care system for patients suspected of serious morbidities which need a closer follow-up.”

Reviewer #2: (No Response)

--------------------

<b>Conclusions</b></br></br>
-Are the conclusions supported by the data presented?</br>
-Are the limitations of analysis clearly described?</br>
-Do the authors discuss how these data can be helpful to advance our understanding of the topic under study?</br>
-Is public health relevance addressed?</br></br>

Reviewer #1: More than the fact that the Test and treat strategy seems to be the best one in the population studies according to authors, they appropriately stress on the big gap of lack of knowledge about Schisto in non endemic areas.

**Answer:** We fully agree with this statement and we hope that article makes clear this central conclussion from our article.

The limitations of analysis is clearly described (in the paragraph "Co-factors that may influence the cost-benefit"). Indeed, they propose a research agenda of further studies, on treatment efficacy, POC tests, etc., in the light of public health challenges.

Reviewer #2: (No Response)

--------------------

<b>Editorial and Data Presentation Modifications?</b></br><br/>
Use  this section for editorial suggestions as well as relatively minor modifications of existing data that would enhance clarity. If the only modifications needed are minor and/or editorial, you may wish to recommend “Minor Revision” or “Accept”.

Reviewer #1: A table comparing the results of the 3 strategies could help the reader at the beginning of the paper.

**Answer:** We include the new table 4 in the discussion section that may respond to the reviewer requirement.

Table 1: please precise that prevalence figures presented are mostly based on parasitic microscopic examinations (to check if possible in the referenced article

**Answer:** Thank you, we included the following foot-note of Table 1:

“These estimations correspond to the observation of eggs in urine or stool samples and mostly coming from children or young adults, whereas the estimations in Europe are from sero-prevalence studies . The real prevalence of the active infection in long-term migrants in Europe remains unknown”

Tables 2 and 3 could be re-arranged for better understanding:

-Table 2 could list Symptoms only (the more specific, if possible) and associated diagnosis procedures (instead of "care ..."). Symptoms could be grouped in genitourinary (S. haematobium), intestinal (S. mansoni) and neurologic Schisto. The diagnosis procedures could be inspired by recommendations  in clinical practice issued from the Spanish Council of Medicine or its equivalent.
All the diagnosis procedures listed do not seem necessary to appear, but the most important are Ultra-sonography and CT.

**Answer:** We arranged Table 2 as suggested. The listed procedures include the basic array of diagnostic procedures to apply in front a given sign or symptom (e.g. hematuria), in spite that most of them would not lead to the diagnostic of a serious or significant morbidity, but contribute to the associated Health System costs of schistosomiasis infection and should be considered in the analysis.

-Table 3: could retain only the pathologies (take off "blood in stools", "hematuria" and other symptoms), which could be hierachied in order of frequency. The estimated occurrence is not referenced for "Female infertility", "stroke" and "pulmonary hypertension". Authors should state that some of these pathologies cannot be ruled out for sure as Schistosomiasis complications. besides to the weighted cost, it could be valuable to add the "Mortality rate" (if we have this data in non endemic countries, unless it should be specified).

**Answer:** We have not precise data about the mortality rate of diferent schistosomiasis complications in developed countries. The focus of our study was not mortality. We introduced the suggested modifications to Table 3 but we moved it to the supplementary material file. We kept the symptoms since they are major contributors of associated costs in form of clinical and complementary procedures.

Table 4: Column "Probable effects", please complete for some factors; column "Recommended research action", please focus on the priority aim of your study i.e. to prevent or detect schisto complications (organ pathologies).

**Answer:** Former table 4 (now table 3) intend to display the effects of gap of knowledge mentioned in the results section, over each of the strategy considered in our costs analysis and how to address them. We expanded the table with some additional information, as follows:

Table 3: Potentially intervening factors, probable effects and recommended actions related to the implementation of schistosomiasis screening program in a non-endemic country

| Factor | Probable effect | Recomended research or evaluation action |
| --- | --- | --- |
| Prevalence of different Schistosoma species | Differential prevalence of Schistosoma-associated morbidites (genitourinary vs. digestive) | Develop more specific diagnostic tools (e.g. PCR-based) |
|  |  |  |
| PZQ efficacy in long-term residents with chronic infection | Limited efficacy of PZQ in preventing morbidities; increase in the number of doses and dosage for long-term infections | Determine the efficacy of PZQ, including recommended dosages, in long-term migrants with morbidities; ascertain the natural history of schistosomiasis infection in non-endemic countries |
|  |  |  |
| Coverage of screening program | Limited coverage of exposed population and hard-to-reach pockets; límit coverage of population at high risk (e.g.new and indocumented migrants) | Include costs of outreach and awareness-raising activities in cost analysis; develop POC tests |
|  |  |  |
| Limited specificity and sensitivity of current diagnostic tests | Increase in the number of unnecessary treatments; false negative results which will result on missed treatment opportunities | Develop more specific and sensitive diagnostic tools, both laboratory and clinical |
|  |  |  |
| Secondary health-related costs | Increase in associated costs of WW | Include additional secondary costs in the cost analysis |
|  |  |  |
| Changes in socio-demographic features of target population | Modulation of associated costs of ST, TT and WW depending on age structure, gender distribution, country of origin and legal status | Monitor exposed population and re-assess cost analysis and screening strategies |
|  |  |  |
| Expanded coverage of schistosomiasis control programs in country of origin | Decrease in prevalence of schistosomiasis among newly arrived migrants | Regularly re-assess prevalence of infection/associated morbidities and the cost analysis of screening strategies |
|  |  |  |
| Cohort effect of screening programs | Relative increase in number of recent infections, general decrease in prevalence of long-term schistosomiasis infection | Regularly re-assess of cost analysis and screening strategies including prevalence of long-term infection |
|  |  |  |

- Inclusion of

  References
  Relatively important references are lacking, as written above, on:
   Complications: Tamarozzi et al. and other European studies
  Prevalence of infection in European countries: Marchese et al.
  strategy of screening: ....

**Answer:** Thank you for your suggestion. These references have been included in the new version of the article as we mention above.

Reviewer #2: (No Response)

--------------------

<b>Summary and General Comments</b></br></br>
Use  this section to provide overall comments, discuss strengths/weaknesses of the study, novelty, significance, general execution and scholarship. You may also include additional comments for the author, including concerns about dual publication, research ethics, or publication ethics. If requesting major revision, please articulate the new experiments that are needed.

Reviewer #1: First I have to say that I am not a Health economist and my scientific background is therefore partially relevant to the subject of the paper. I could not expertise the calculation of the cost estimates and hope that other reviewers will do it. However, some assumptions made for building the various scenarii, especially the first one which acts as basis (spontaneous evolution) have raised my interest.
This paper do not raise the question of a global health burden in the host country, but it opens the persepctive of Schisto screening being a part of an extended health assessment for migrants.

Reviewer #2: This paper investigates an important reach gap regarding the costs of schistosomiasis screening programs in non-endemic countries. I have the following comments/suggestions

Major comments

I understand that the authors have looked at a lot of scenarios. However, overall I found that results very hard to follow. In particularly the averted costs need to be made much clear – potential have results tables rather than just figures (even if some of the scenarios are just reported in the appendix). It needs to be made clearer what is driving these averted costs.

**Answer:** To make the article clearer and more easy-reading, we reshaped the article as we mention above, we moved figure 3 and former table 3 to the supplementary material section, we rounded the costs estimates numbers and we kept the most essential results of the cost-estimation in the text. Additional tables in the supplementary material show the full array of averted costs under diferent scenarios.

Results to the above expand on the thresholds were the optimum intervention changes.

**Answer:** We included the thresholds of prevalence for the neutral averted costs at the results section:

Page 18, lines 322-326: “The PT strategy would match the two-dose TT-2 strategy at prevalence around 25% (see Figure 1). Considering the costs-per-infected person the cost equivalence of , PT, TT-2 and TT-1 with the WW strategy at 40% of costs (970€) are estimated at prevalence of 14%, 10% and 9%, respectively (see Figure 2).”

Page 20, lines 347-349: “The neutral balance (zero averted costs) was estimated at 15% prevalence, 50% of treatment efficacy and around 50% of estimated morbidities-associated costs.”

The Estimated Proportion of occurrence (%) within Table 3 needs to have a reference number that corresponds to the reference list. In the legend it just says WHO for most of these.

**Answer:** We included/excluded the necessary references as requested.

I do not see how a treatment efficiency of 100% could be reasonable. Do you have any references to support this

**Answer:** We make in clear that this is a theoretical but unexpected scenario which acts as a reference point in the following sites:

Materials and Methods section, Page 9, lines 198-201: “Therefore, we considered different reasonable scenarios of average efficacy of treatment ranging from 40% to 100%. Even though a 100% cure is considered as a reference point, not a realistic expectative.”

Material and Methods section, page 16, lines 262-265: “In other words, this estimate represents the maximum amount of resources which could potentially be averted if the disease were detected in time, the symptoms treated before the long-term complications appeared and under the theoretical assumption of 100% effectiveness of treatment”

Results section, page 20, lines 343-347: “It will be seen that in almost all scenarios considered the TT strategy implies net savings relative to the WW strategy, which rise to €58,6 milions assuming 30% prevalence in our study area , 100% treatment efficacy (admittedly unexpected) and 100% potentially averted costs (upper estimate bounds).”

We included the following new references and statement, supporting these appretiations:

28.	Zwang J, Olliaro PL. Clinical efficacy and tolerability of praziquantel for intestinal and urinary schistosomiasis-a meta-analysis of comparative and non-comparative clinical trials. PLoS Negl Trop Dis. 2014;8:e3286. doi: 10.1371/journal.pntd.0003286.

30.	Cucchetto G, Buonfrate D, Marchese V et al. High-dose or multi-day praziquantel for imported schistosomiasis? A systematic review. J Travel Med. 2019;26:taz050. doi: 10.1093/jtm/taz050.

Results section, page22, lines 392-396: “The optimal treatment efficacy cut-offs are still a matter of debate, including dosage regimen, and multicenter clinical trials in non-endemic areas are still needed [30], but it seems clear that a single dose does not reach 100% efficacy in the chronic phase [28]. “

What assumption were made regarding the diagnostic sensitivity

**Answer**: Ideally, the TT strategy is based on the availability of enough sensitive and specific screening methods, which doesn’t exist in current practice. We stated this in the assumptions of the modelling study and consequently, one of the major gaps to be filled in is the development of such screening tools: new tests, POC and/or the inclusion of clinical checking during the screening process. We included the following statements in the present version to make it clearer:

Methods: page 12, lines 239-242 “3) a test-and-treat strategy (TT), whereby a serology-based test with high sensitivity and specificity test would be carried out on all Schistosoma-exposed individuals, with all those testing positive being subsequently treated with a standard dosage of PZQ”

Discussion, page 27, lines 516-520: “However, the results of the TT cost analysis are based on the assumption of a highly sensitive and specific screening tool, which is not the case for the current serology-methods. Serology test is the recommended screening method in non-endemic countries [10], but the value to detect active infections needs to be better ascertained. “

In many places you talk about the “cost/benefit ratio” – I believe this is confusing as my understanding is that this is meant to be a costing study and not a cost-benefit analysis? If it is meant to be a economic evaluation (i.e cost-benefit analysis) the methods needs to be expanded upon. It needs to be made clearer what criteria you are using to say an strategy is optimum.

**Answer:** We correct and substitute cost/benefit by cost-analysis whenever necessary.

I found the Costs associated with PT and TT strategies hard to follow and suggestion these are summarized in a table. Related to this a Table summarizing the different strategies could be helpful

**Answer:** See our previous comments about the general ammendments introduced that would make easier the reading the article.

Minor comments

In many places you talk about the “cost/benefit ratio” – I believe this is confusing as my understanding is that this is meant to be a costing study and not a cost-benefit analysis.

**Answer:** We have revised and corrected this issue all along the manuscrit. We refer now to cost analysis.

Abstract; I found the following sentence hard to understand – consider rephasing: “The difference in averted costs between test-and-treat and watchful waiting strategies ranges from nearly €60 million in scenarios of 30% prevalence, 100% associated costs and 100% treatment efficacy, to a neutral cost/benefit ratio when these parameters are halved.”

**Answer:** We rephrased this sentence as follows : “The difference in averted costs between test-and-treat and watchful waiting strategies ranges from nearly €60 million in high prevalence and treatment efficacy scenarios, to a neutral costs ratio when these parameters are halved”

Table 3: Some of the values seem to have 4 decimal places – would consider rounding.

**Answer:** numbers have been rounded

I would consider not having abbreviations for the different strategies. It may save words but makes it much harder to read (this is just my opinion and  is the authors decision)

**Answer:** We spared a certain number of abbreviations since we do not consider any moret he PT-2 (presumptive treatment with two dosages) in our study. However, we have the impression that unless we use these abbreviations the text would be more hard to read with a substantial overwording.
