# Supplementary material for: Schistosomiasis screening in non-endemic countries from a cost perspective: Knowledge gaps and research priorities. The case of African long-term residents in a Metropolitan Area, Spain
Source: PLoS Negl Trop Dis. 2023 Apr 4;17(4):e0011221. doi: 10.1371/journal.pntd.0011221 (PMC10104311; doi:10.1371/journal.pntd.0011221)
Supplement: Attachment — Submitted filename: Responses to reviewers.docx [file pntd.0011221.s004.docx]

**Responses to reviewers and point by point modifications introduced in the article**

Reviewer's Responses to Questions

<b>Key Review Criteria Required for Acceptance?</b></br></br>
As you describe the new analyses required for acceptance, please consider the following:</br></br>

<b>Methods</b></br></br>
-Are the objectives of the study clearly articulated with a clear testable hypothesis stated?</br>
-Is the study design appropriate to address the stated objectives?</br>
-Is the population clearly described and appropriate for the hypothesis being tested?</br>
-Is the sample size sufficient to ensure adequate power to address the hypothesis being tested?</br>
-Were correct statistical analysis used to support conclusions?</br>
-Are there concerns about ethical or regulatory requirements being met?</br></br>

Reviewer #1: same as in the first review

Reviewer #2: (No Response)

--------------------

<b>Results</b></br></br>
-Does the analysis presented match the analysis plan?</br>
-Are the results clearly and completely presented?</br>
-Are the figures (Tables, Images) of sufficient quality for clarity?</br></br>

Reviewer #1: same as in the first review

Reviewer #2: (No Response)

--------------------

<b>Conclusions</b></br></br>
-Are the conclusions supported by the data presented?</br>
-Are the limitations of analysis clearly described?</br>
-Do the authors discuss how these data can be helpful to advance our understanding of the topic under study?</br>
-Is public health relevance addressed?</br></br>

Reviewer #1: same as in the first review

Reviewer #2: (No Response)

--------------------

<b>Editorial and Data Presentation Modifications?</b></br><br/>
Use  this section for editorial suggestions as well as relatively minor modifications of existing data that would enhance clarity. If the only modifications needed are minor and/or editorial, you may wish to recommend “Minor Revision” or “Accept”.

Reviewer #1: YES : to add a sub-tittle to the article’s title : «Schistosomiasis screening in non-endemic countries from a cost perspective : knowledge gaps and research priorities. The case of African long-term residents in the Metropolitan Area, Spain. »
I suggest to add the same precision in the Objectives of the Abstract

**Response: following the reviewer suggestion, we agree to include this subtitle in the new revised version of the artilce**

Reviewer #2: (No Response)

--------------------

<b>Summary and General Comments</b></br></br>
Use  this section to provide overall comments, discuss strengths/weaknesses of the study, novelty, significance, general execution and scholarship. You may also include additional comments for the author, including concerns about dual publication, research ethics, or publication ethics. If requesting major revision, please articulate the new experiments that are needed.

Reviewer #1: I read the answers of the Authors.
I agree with most of the modifications they did.
I agree with the stress made by the authors on the characteristic of the migrant population studied, i.e. the fact that more than half have been living there for more than 10 years .
I therefore suggest to add a sub-tittle to the article’s title : «Schistosomiasis screening in non-endemic countries from a cost perspective : knowledge gaps and research priorities ».The case of African long-term residents in the Metropolitan Area, Spain.
I suggest to add the same precision in the Objectives of the Abstract.

Answer: We included the suggested subtitle. In addtion, we introduced the follwing ammendments in the Abstract:

Lines 60-61 (background): “Undetected infection may lead to serious long-term complications with an associated high cost for public healthcare systems ***especially among long-term migrants***.”

Lines 62-64 (objectives): “To evaluate from a health economics perspective the introduction of schistosomiasis screening programs in non-endemic countries ***with high prevalence of long-term migrants****”*

Lines 82-84 (Conclusions): “...but important knowledge gaps should be addressed for a more accurate estimations ***among long-term migrants***.”

Reviewer #2: The authors have comprehensively addressed my comments. I have the flowing minor comments

There are a number of small typos (particularly around million), extra “.” and “,” and errors in the bullet point numbering (line 476. The manuscript needs another in-depth proofread.

**Answer: We revised the manuscript carefully. We expect to have solved all these typos.**

Avoid saying “cost-effective strategy” in the conclusions– as you have not done a cost-effectiveness analysis. Maybe say cost-optimum

**Answer: We chagned cost-optimum instead of cost-effective in lin 631 (end of de discussion paragraph).**

There are a number of small typos (particularly around million), extra “.” and “,” and errors in the bullet point numbering (line 476. The manuscript needs another in-depth proofread.

**Answer: We revised the manuscript carefully. We expect to have solved all these typos.**

I would avoid having gridline in the fingers – for figure 2 it is hard to tell which line is a strategy vs a gridline

**Answer: We have rebuilt figures with higher quality and resolution, including colors and avoiding gridlines to make them clearer and more readful.**

“Not all morbidity-associated care procedures are indicated for any given patient (the 100%-cost scenario” I found this unclear. Please can you clarify more clearly what the % Scenarios for the WW strategy and morbidities-associated costs are (i.e. what is meant by 100% of the costs being incurred here). A table that summarizes the strategies/ scenarios that corresponds to the main figures might be helpful – particularly as the treatment efficacy range and treatment costs for long-term morbidities range are both 40%-100%.

**Answer: We erased the “(the 100%-cost scenario)” in line 191 of the Materials and Methods section. The table summarizing the strategies-scenarios that corresponds to the main figures are included as supplementary material.**

What are the treatment efficacy assumptions within the two main figures?

**Answer: Figure 1: As state in the figure legend “Total projected cost for each strategy at different prevalence rates and for the WW strategy, at five percentages of the maximum cost of treatment of associated morbidities”, each WW-line represents an specific esitmated associated care and diagnostic costs for the WW (from 100% to 40%), in total five cut-offs (100%, 85%, 70%, 55% and 40%). To make it clearer we introduced the following foot-note in Figure 1.**

**“The % displayed in each WW associated costs lines correspond to the specific estimation of diagnostic and treatment costs of schistosomiasis associated morbidities under this strategy, from 100% to 40%”**

**The same answer applies for Figure 2: We included the same footnote.**

Add abbreviations to the figure legends.

**Answer: We included the abbreviations to the figure legends as foot-notes: WW, PT and TT as suggested**

“The neutral balance (zero averted costs)” I think saying zero averted costs here might confuse the readers – maybe say it is cost neutral.

**Answer: Thank you for this suggestion. We erased “(zero averted costs)” from the text in line 352.**

Make sure the terms are consistent. For example, in Table 4 it says Wait and watchful strategy vs watchful waiting strategy in the text

**Answer: We revised the manuscript for consistency and we expect to have solved all these issues. Thank you for letting us know.**

**Additionally, we reshaped and reformated Figures 1 and Figures 2 to make them clearer, and we introduced an additional reference published during the revision period of this article: “Zammarchi L, Botta A, Tilli M, Gobbi F, Bartoloni A, Boccalini S. Presumptive treatment or serological screening for schistosomiasis in migrants from Sub-Saharan Africa could save both lives and money for the Italian National Health System: results of an economic evaluation. J Travel Med. 2022:taac140. doi: 10.1093/jtm/taac140.” Now reference 46.**

**With this regard, we introduced the following additional statements in the Discussion section:**

**Lines 508-512: “Our results are in agreement of a recently work published in Italy [46].** However, **the results from this previous work and this present article** are based on the assumption of a highly sensitive and specific screening tool, which is not the case for the current serology-methods.”

**Lines 537-542: “**These contrasting assessments underscore the unknowns we have identified regarding the natural history of Schistosoma infection in non-endemic countries (for instance, the efficacy of PZQ for long-term infections), **which have not been taken into account in previous works and the particular clinical profile of long-term exposed migrants [20,46].”**

**Finally we included an additional affiliation to the last autor (affiliation 18).**
